# Isolation of a novel *Saccharophagus* species (Myt-1) capable of degrading a variety of seaweeds and polysaccharides

**DOI:** 10.1002/mbo3.10

**Published:** 2012-03

**Authors:** A Sakatoku, M Wakabayashi, Y Tanaka, D Tanaka, S Nakamura

**Affiliations:** Graduate School of Science and Engineering, University of ToyamaToyama 930-8555, Japan

**Keywords:** Alginate lyases, agarases, cellulases, degradation, *Saccharophagus*, seaweeds

## Abstract

A bacterial strain, Myt-1, was isolated in Toyama Bay in Toyama Prefecture, Japan. Myt-1 was capable of reducing the thalli of various seaweed species to single cell detritus particles. A 16S rDNA homology search revealed that the closest relative of Myt-1 was *Saccharophagus degradans* 2–40 (CP000282; 100% similarity), which was first isolated in Chesapeake Bay in Virginia, USA. The Myt-1 strain was capable of degrading more than 10 polysaccharides, almost all of which were also degraded by *S*. *degradans* 2–40. Analyses of alginase gene DNA sequence homology, DNA–DNA homology, and zymogram analysis of obtained polysaccharidases suggested that Myt-1 was a new species of *Saccharophagus*. Thus, Myt-1 is only the second species in this genus, which has contained only one strain and species since 1988, and was tentatively designated *Saccharophagus* sp. Myt-1. Myt-1 has considerable potential for reducing the volume of seaweed wastes, and for producing functional materials from seaweed substrate.

## Introduction

Total global production of seaweeds and other aquatic plants in 2008 was approximately 1.1 million tons harvested from the ocean and 15.8 million tons produced by aquaculture (FAO 2010). Associated with this increase in seaweed production has been an increase in the quantities of seaweed residues that are produced by industry (Fei et al. 1999); for example, more than 200,000 tons of Wakame (*Undaria pinnatifida*) is produced annually in Japan (Fujii and Korenage 2000). Another reason for the global increase in the quantity of seaweed waste that is produced annually is due to the increase in drifting seaweeds resulting from the eutrophication of coastal waters (Morand and Briand 1996; Morand and Merceron 2005). Because most of this seaweed waste is interned in landfills or incinerated, the ability to either use or reduce the quantities of this harvested seaweed waste is receiving increased attention around the world.

Seaweeds, specifically, brown, red, and green macro algae, produce large quantities of polysaccharides, such as alginic acid, agar, fucoidan, laminarin, cellulose, and carrageenan. These polysaccharides have a variety of applications, including antitumor agents (Fukahori et al. 2008), antioxidants (Rocha de Souza et al. 2007), antiinflammatory agents, anticoagulants (Li et al. 2008), food ingredients, cosmetics, industrial products (Dhargalkar and Pereira 2005), and biofuel production (Hom et al. 2000). In addition, the degradation of seaweed thallus fragments has been used to produce single cells or protoplasts for aquaculture diets (Uchida et al. 1997; Camacho et al. 2004).

Numerous studies have examined the isolation and characterization of polysaccharide-degrading bacteria from seawater (Uchida et al. 1995; Kawamoto et al. 2006), seaweed surfaces (Aoki et al. 1990; Wang et al. 2006; Kim et al. 2008), marine sediments (Ohta et al. 2004; Uchimura et al. 2010), and decomposing seaweeds (Jonnadula et al. 2009; Tang et al. 2009). The most efficient degrader of complex polysaccharides identified to date appears to be *Saccharophagus degradans* 2–40 (Ekborg et al. 2005). *Saccharophagus degradans* 2–40 was first isolated from decaying salt marsh cordgrass collected in the Chesapeake Bay estuary (Andrykovitch and Marx 1988). Because strain 2–40 is capable of degrading more than 10 complex polysaccharides, including agar, alginate, carrageenan, carboxymethyl (CM) cellulose, chitin, β-glucan, laminarin, pectin, pullulan, starch, and xylan (González and Weiner 2000; Weiner et al. 2008), this species is likely to play an important role in the marine carbon cycle. However, to the best of our knowledge, only one species and strain have been assigned to genus *Saccharophagus* (i.e., *S. degradans* 2–40) to date. In this study, we isolated and characterized a novel species of *Saccharophagus* that was capable of degrading different kinds of seaweed and their component polysaccharides.

## Materials and Methods

### Isolation of seaweed-degrading bacteria

Marine sediments from the fishing ports of Horioka and Yokata in Toyama Prefecture, Japan, were collected in May–July 2010. After being transported to the laboratory in iceboxes, the samples were prepared for analysis. The primary culture medium used in this study consisted of artificial seawater (ASW) culture medium (ASW 800 mL, NH_4_NO_3_ 1 g, K_2_HPO_4_ 0.02 g, yeast extract 0.5 g, and deionized water 200 mL, pH 7.8) (Higashihara et al. 1978), with marine Art SF-1 (Tomita Pharmaceutical Co. Ltd., Tokushima, Japan) used as the ASW in the ASW culture medium. To isolate bacteria capable of degrading seaweed, 1 g (wet weight) of sediment was transferred to a conical beaker containing 100 mL of ASW and dried Wakame thallus fragments (0.25% w/v; Muroya Co. Ltd., Toyama, Japan), and incubated at 25°C on a rotary shaker at 140 rpm (Bioshaker BR-180LF; Taitec Corp., Saitama, Japan) for seven days. When degradation of Wakame thallus fragments was observed, a loop-full of the culture medium containing the cultured bacteria was spread on ASW agar plates (1.5% w/v). Any colonies were then picked from the plates and used to inoculate ASW liquid medium containing Wakame thallus fragments (0.25% w/v) with shaking for two–three days. Any bacterial clones that were observed to degrade seaweed fragments were then preserved on ASW agar slants or plates for use in this study. *Saccharophagus degradans* 2–40^T^ (ATCC® 43961™) was obtained from the American Type Culture Collection (Manassas, VA).

### Degradability of seaweeds

The degradability of seaweeds was examined using ASW containing 0.25% (w/v) dried brown alga (Wakame), dried green alga (*Ulva* sp.), or dried red alga (*Gelidium* sp). The green and red algae were collected along the Toyama Bay coast. The ASW samples containing the algal substrates were inoculated with bacterial cells at approximately 10^6^ cells·mL^−1^ and incubated at 25°C with shaking at 140 rpm (Bioshaker BR-180LF). Seaweed degradation was identified with the naked eye and examined under a phase-contrast microscope (Olympus BX-51, Tokyo, Japan). After the filtration of culture medium through a nylon mesh (pore size: 100 μm; PP-100N; Kyoshin Rikoh Inc., Tokyo, Japan), the densities of single cells or particles produced by bacterial degradation was estimated under a phase contrast microscope using a counting chamber.

### Morphological and physiological analysis of the microorganism

The morphology and motility of the bacteria were first observed by a phase-contrast microscope (Olympus BX-51). Samples were then prepared for examination under an electron microscope by negatively staining them with 1% (w/v) phosphotungstic acid and then observing them under a JEM-100 SX electron microscope (JEOL, Tokyo, Japan). Micrographs were taken at an accelerating voltage of 80 kV. Gram staining was performed using a FAVOR-G SET-S kit (Nissui Pharmaceutical Co. Ltd., Tokyo, Japan). Color changes of the colonies were observed by culturing on plates of Marine Agar 2216 (Difco; Becton, Dickinson and Company, Sparks, MD).

### rDNA and alginate lyase gene sequencing

Genomic DNA was extracted from a colony of Myt-1, and 16S rDNA was amplified by the polymerase chain reaction (PCR) using the eubacterial universal primers 27f (5′-AGAGTTTGATCCTGGCTCAG-3′) and 1525r (5′-AAAGGAGGTGATCCAGCC-3′). Each PCR sample contained 1 × Ex Taq buffer, 200-μmol·L^−1^ dNTP mix, 0.25-μmol·L^−1^ of each primer, 0.5 U Ex *Taq* HS (*Taq* DNA polymerase; Takara Bio Inc., Shiga, Japan). PCR reactions were performed using a thermal cycler (Takara PCR Thermal Cycler Dice, Takara Bio Inc.) with an initial denaturation step of 94°C for 3 min, followed by 35 amplification cycles consisting of denaturation at 94°C for 60 sec, annealing at 60°C for 60 sec, and elongation at 72°C for 60 sec. The reaction was terminated with a terminal elongation step of 72°C for 3 min followed by cooling at 4°C. The amplified products were purified with a QIAquick PCR purification kit (Qiagen K. K., Tokyo, Japan) before being sequenced directly using a BigDye Terminator v3.1 cycle sequencing kit (Applied Biosystems, Foster City, CA), an ABI Prism 3130xl genetic analyzer (Applied Biosystems), and the primers, r1L, r2L, r3L, r4L, f1L, f3L, and 926f (Tanaka et al. 2010). The homology sequences of the isolates were then searched by the BLAST program on the NCBI website (http://www.ncbi.nlm.nih.gov). A phylogenetic tree was constructed using the neighbor-joining algorithm (Saitou and Nei 1987) and sequence data of related species were obtained from the GenBank.

The gene encoding alginate lyase was then amplified from the genomic DNA of strain Myt-1 by PCR. Marine broth medium (Difco Marine Broth 2216; Becton, Dickinson and Company) was used as the culture medium for the Myt-1 cells from which the genomic DNA was extracted. Two primer sets, Sde2873f (ATGAGAAGTGTATTGCTTCCA), Sde2873r (TTAAAAATTGGTGGCAGAGCC), Sde2547f (ATGACATTCATCAAAATCATGG), Sde2547r (CTAGTCGTGAGTATGCGTAAG), were designed from two putative *S. degradans* alginate lyase genes, *alg7A* and *alg7B*. The amplified products were then purified and sequenced as for the 16S rDNA sequencing. The full lengths of the alginate lyase genes were sequenced by the primer walking (Szybalski 1993) and inverse PCR (Howard et al. 1988) methods. Restriction digests were performed using 3 μg of genomic Myt-1 DNA treated with 40 U of *Sau*3AI. One nanogram of digested DNA was then circularized with a Rapid DNA Dephos and Ligation Kit (Roche, Manheim, Germany). The ligated sample was then treated with an equal volume of phenol–chloroform–isoamylalcohol (25:24:1) before the aqueous phase was removed and the DNA was precipitated with ethanol and collected by centrifugation. The entire genes were amplified by nested PCR. Each of the two primer sets was designed from the partially sequenced alginate lyase genes, *algMytA* and *algMytB*. Primer sequences for *algMytA* were as follows: 1st-f, CTATGGTTCGATGCTGCCAGCACCT; 1st-r, CCGCCAAATGGTAAGCACTTGCGAA; 2nd-f, CGATGCTGCCAGCACCTTTGGTTAC; 2nd-r, TGGTAAGCACTTGCGAACAACGGTT; and for *algMytB* were as follows: 1st-f, AGTGGTTACGACGACTCCGACGATT; 1st-r, CCACCATGGCTAATTTGAACCCAGT; 2nd-f, CGACGACTCCGACGATTGGATGTAT; 2nd-r, GCTAATTTGAACCCAGTTCTGGTTG. The first amplification reaction was performed in a 40 μL first PCR reaction mixture containing a 1 × PCR buffer for KOD FX (Toyobo, Osaka, Japan), 0.3 μmol·L^−1^ for each primer, 0.4-mmol·L^−1^ dNTPs, 1 U of KOD FX (Toyobo), and 1 ng of precipitated DNA. PCR amplification was performed using an initial denaturation step of 94°C for 2 min, followed by 30 cycles at 98°C for 10 sec, 50°C for 30 sec, and 68°C for 4 min, before the temperature was maintained at 4°C. The second amplification reaction was performed in a 40-μL PCR reaction mixture containing 1 × PCR buffer for KOD FX, 0.2 μmol·L^−1^ for each primer, 0.4-mmol·L^−1^ dNTPs, 1 U of KOD FX, and 1 μL of amplified product from the first PCR. This second amplification reaction was performed using the same program as the first PCR. The resulting PCR products were also purified and sequenced as described for the 16S rDNA. Nucleotide and deduced amino acid sequence analyses, open reading frame (ORF) searches, multiple sequence alignments, molecular mass, and isoelectric point calculations were performed using Genetyx Ver.8 software (Genetyx, Tokyo, Japan). A database homology search was performed using the BLAST program on the NCBI website (http://www.ncbi.nlm.nih.gov).

The G + C content of genomic DNA was measured by TechnoSuruga Laboratory Co. Ltd. (Shizuoka, Shizuoka, Japan) using a high performance liquid chromatography as described by Katayama-Fujimura et al. (1984) and Nishijima et al. (2009). The DNA–DNA hybridization analyses were also performed at the TechnoSuruga Laboratory Co. Ltd. by the fluorometric hybridization method on microdilution plates (Ezaki et al. 1989).

### Growth and polysaccharide-degrading activities

Strain Myt-1 was cultured in 100 mL ASW containing dried Wakame thallus fragments (0.2% w/v). Myt-1 cells were inoculated at concentrations of approximately 10^6^ cells·mL^−1^ and incubated at 25°C with shaking at 140 rpm for seven days. Bacterial growth was estimated by viable bacterial counts, which were determined by plotting CFU·mL^−1^ of serial dilutions on agar plates as a function of time. After centrifugation of aliquots at 12,000 × *g* for 15 min at 4°C, the concentration of reducing sugars in the supernatants were measured by the method of Somogyi–Nelson (Somogyi 1952; Nelson 1994). The polysaccharides-degrading enzyme activities in the supernatants were determined by degradation of the following polysaccharides: sodium alginate (80∼120 cP), pectin, pullulan, starch (Wako Pure Chemical Industries, Ltd., Osaka, Japan), CM cellulose (Sigma-Aldrich, St. Louis, MO), Bact Agar (Difco), agarose (Nippongene, Tokyo, Japan), chitin (Tokyo Chemical Industry Co. Ltd., Tokyo, Japan), fucoidan (from *Fucus vesiculosus*; Sigma-Aldrich), laminarin, carrageenan, polygalacturon, inulin, glycogen (Nacalai Tesque, Kyoto, Japan), xylan (SERVA Electrophoresis GmbH, Heidelberg, Germany). A reaction mixture containing 100 μL of the supernatant and 300 μL of 0.2% (w/v) substrate in 20-mmol·L^−1^ Tris-HCl (pH 8.0) was incubated at 30°C for 1 h. The reducing sugars released into the medium were measured by the method of Somogyi–Nelson. Concentrations of these reducing sugars were determined using D-glucose as a standard. One unit of enzyme activity was defined as the amount of enzyme required to produce 1.0 μg·mL^−1^·h^−1^ of reducing sugars (glucose equivalents) (Uchida 1995).

### Zymogram analysis by sodium dodecyl sulfate-polyacrylamide gel electrophoresis (SDS-PAGE)

Strain Myt-1 and *S. degradans* 2–40 were cultured in ASW medium containing Wakame (0.25% w/v) at 25°C for three days. The culture medium was centrifuged at 13,000 *g* for 15 min at 4°C and the supernatant was filtered using a polycarbonate membrane filter (pore size: 0.2 μm; Advantec, Toyo Roshi Kaisha, Ltd., Tokyo, Japan). The filtrate was concentrated ca. 200-fold using an ultra filtration membrane (Amicon Ultra-15, 10 000 nominal molecular weight limit; Millipore Corporation, Bedford, MA). The concentrated filtrates or pellets from the aforementioned centrifugation step were mixed with the sample solution of Laemmli (1970) and boiled for 5 min. SDS-PAGE was performed on 7.5% acrylamide gels. The gels were stained for proteins using Quick Blue Staining Solution (Bio Dynamics Laboratory Inc., Tokyo, Japan) and the molecular mass of the resulting bands was estimated using high- and low-range molecular mass standards (Bio-Rad Laboratories, Inc., Tokyo, Japan), which included myosin (200 kDa), β-galactosidase (116.3 kDa), phosphorylase b (97.4 kDa), serum albumin (66.2 kDa), ovalbumin (45 kDa), carbonic anhydrase (31 kDa), trypsin inhibitor (21.5 kDa), and lysozyme (14.4 kDa). Protein concentrations were measured with a Pierce bicinchoninic acid protein reagent kit (Interchim, Montluçon, France).

Alginate lyase was identified by zymogram analysis using the method of Pecina and Paneque (1994) with minor modifications. Briefly, after SDS-PAGE, gels containing 0.2% (w/v) sodium alginate were washed three times with Milli-Q water for 15 min at room temperature. The proteins in each gel were then renatured by incubation in renaturation buffer (50-mmol·L^−1^ Tris-HCl (pH8.2), 1% (w/v) casein, 2-mmol·L^−1^ EDTA, 0.01% NaN_3_) for three 30-min periods with gentle shaking at 4°C. The gels were then incubated in 10-mmol·L^−1^ phosphate buffer (pH 7.5) containing 0.2-mol·L^−1^ NaCl, 0.2-mol·L^−1^ KCl, and 0.01% NaN_3_ for 2 h at room temperature with gentle shaking. The gels were then stained by gentle shaking in a solution of 10% (w/v) cetylpyridinium chloride for 20 min at room temperature. The activity of alginate lyase was visualized as a clear zone and photographed on a black background (Fig. 5b).

To detect cellulase activity, we used SDS polyacrylamide gels containing 0.1% (w/v) CM cellulose. Following electrophoresis, gels were washed with Milli-Q water for 15 min before incubation at room temperature in 0.1 mol·L^−1^ succinic acid buffer (pH 5.8) for 2 h. The gels were then submerged in 0.01% (w/v) Congo red solution for 10 min and washed with 1-mol·L^−1^ NaCl until clear bands were visible (Schwarz et al. 1987). To detect agarase, gels containing 0.02% (w/v) agar were subjected to SDS-PAGE. After electrophoresis, gels were incubated in 20-mmol·L^−1^ Tris-HCl buffer (pH8.0) at room temperature for 2 h, after which after the gel was stained by gentle shaking in an iodine–potassium iodide solution (1% (w/v) iodine, 2% (w/v) potassium iodide) for 2 h at room temperature before washing with Milli-Q water.

## Results

### Isolation of seaweeds-decomposing bacterial strain Myt-1

**Figure 1:**
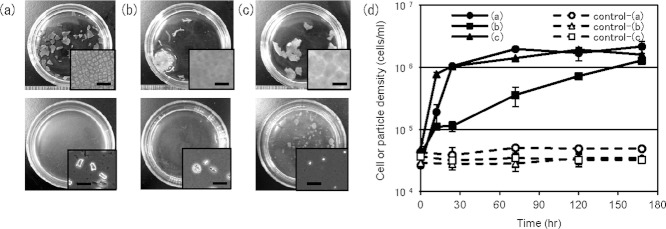
Degradation of seaweed thallus fragments from three types of algae by the bacterial strain Myt-1, showing fragments before (top row) and seven days (168 h) after inoculation (bottom row). Insets show enlarged views of thallus flakes before inoculation (top row), and thallus tissue that has been degraded into single cells, or fine particles (bottom row); scale bars show 10 μm. (a) Brown alga (Wakame; *Undaria pinnatifida*); (b) red alga (*Gelidium* sp.); (c) green alga (*Ulva* sp.). (d) Changes in cell or particle (≤100 μm) densities resulting from thallus degradation by strain Myt-1. Controls in graph (d) uninoculated samples.

We isolated 50 bacterial strains that were capable of decomposing seaweed thalli from marine sediments collected at a site in Toyama Bay, Japan. Each isolate was cultivated in a medium containing Wakame thalli fragments as the sole carbon source. Of the isolates examined, strain Myt-1 degraded substrates most rapidly and into the finest particles after one day at 25°C (Figs. 1a, d). Before inoculation with Myt-1 cells (Fig. 1a upper row) and the control, that is, no Myt-1 inoculation (data not shown), the Wakame thallus fragments were intact, and abundant multicellular sheets were observed on the surface of the fragments under a microscope. After incubation with Myt-1 cells for at least one day, most of the Wakame thallus fragments were degraded into numerous, fine, rectangular-shaped algal particles (Fig. 1a (lower row)). The average dimensions of these algal particles, which have been referred to previously as single-cell detritus particles (Uchida 1997; Camacho et al. 2004), measured approximately 10.3 × 5.7 × 5.1 μm. Strain Myt-1 was also capable of decomposing other seaweed species, red alga (*Gelidium* sp.) and a green alga (*Ulva* sp.) (Fig. 1b, c, d), but the time required for Myt-1 to degrade the red and green algae was longer than that required to degrade Wakame. In addition, the degraded products of the green alga were finer than those obtained from Wakame. No such degradation was observed in the control samples (Fig. 1d).

**Figure 2:**
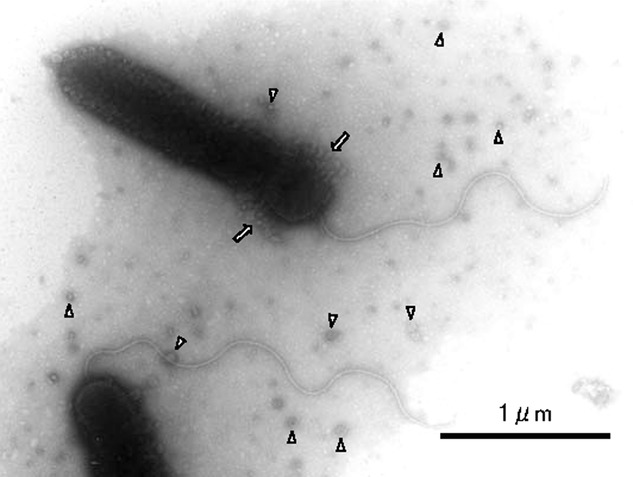
Electron micrograph of negatively stained Myt-1 cells. Cells were rod shaped and had a single polar flagellum. Numerous small vesicles were observed on the cell surface and in the medium (arrowheads) adjacent to the cell. Vesicles on the cell surface were shed from the near posterior end of the cell (arrows). Bar: 1 μm.

Strain Myt-1 is a motile Gram-negative bacterium. The rod-shaped cells were 0.4-μm wide and 1.8-μm long (on average) and had a single polar flagellum (Fig. 2). Numerous small vesicles were present on their cell surfaces and in the culture medium. These vesicles were deciduous and were shed from the cell surface near the rear end (flagellated side) of the cell. In addition, numerous vesicles were observed in the vicinity of the cell. Circular colonies were formed two days after inoculation and they were concave due to the degradation of the agar in the plates. Colonies were initially off-white in color, changing to black after four days of culture on Marine agar plates.

The 1489-bp 16S rDNA sequence of strain Myt-1 was used to identify the strain to species. The sequence showed 100% similarity with *S. degradans* (GenBank accession number CP000282) strain 2–40, described by Ekborg et al. (2005). The 16S rRNA gene sequence of Myt-1 was deposited in GenBank under accession no. AB566414. The G + C content of strain Myt-1 was 45.9 mol% and that of strain 2–40 was 45.8 mol% (Weiner et al. 2008). DNA–DNA hybridization results showed that strain Myt-1 shared 65–68% DNA–DNA homology with strain 2–40.

**Figure 3:**
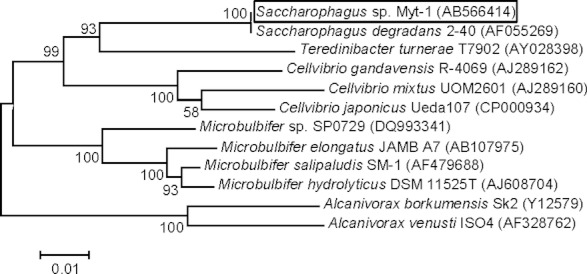
Phylogenetic tree based on partial 16S rDNA sequences of Myt-1(*Saccharophagus*) and related species. Numbers at nodes indicate bootstrap values as a percentage of 1000 replicates. Bar shows 0.01 substitutions per nucleotide position.

A phylogenetic tree based on partial 16S rRNA gene sequences showed the relationship between strain Myt-1 and other related species obtained from GenBank (Fig. 3). In addition to *S. degradans* 2–40, GenBank sequences for *Cellvibrio* spp. and *Teredinibacter* spp. were similar to those of strain Myt-1.

### Growth and quantification of reducing sugars

**Figure 4:**
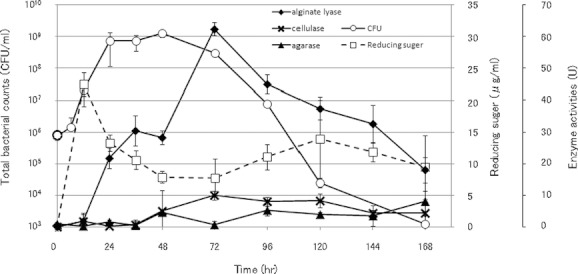
Extracellular polysaccharide-degrading activities associated with bacterial cell growth in artificial seawater containing Wakame thallus fragments. (○) Average growth rate and (□) quantity of reducing sugars produced in the culture supernatants are shown. Polysaccharidase activities of (♦) alginate lyase, (×) cellulase, (▴) agarase were measured at each incubation time and are expressed in units (U). Units were equivalent to the quantity of enzyme required to produce 1 μg·mL^−1^·h^−1^ of reducing sugars (glucose equivalents). Values are means ± SE, *n* = 3.

The production of reducing sugars by strain Myt-1 cultured in ASW containing 0.2% (w/v) Wakame thalli fragments was examined (Fig. 4). The cell density of Myt-1 increased rapidly after inoculation and reached 1.0 × 10^9^ CFU·mL^−1^ 24–48 h after inoculation, before decreasing rapidly to 2.5 × 10^4^ CFU·mL^−1^ after 72 h. At ca. 22.5 μg·mL^−1^, production of reducing sugars by strain Myt-1 was highest 12 h after incubation, before decreasing to approximately 7 μg·mL^−1^ within 72 h, when levels almost plateaued for another 72 h. Enzyme activities in the culture supernatant of strain Myt-1 were tested against several polysaccharides (i.e., alginate, cellulose, and agar) (Fig. 4). Alginate lyase demonstrated the highest levels of activity (60 U at 72 h after inoculation), followed by cellulase (10 U at 72 h), and agarase, which exhibited the lowest activities of <0.8 U in the experiment period.

*Saccharophagus degradans* strain 2–40 has been reported to degrade at least 10 polysaccharides, including agar, alginate, chitin, cellulose, fucoidan, laminarin, pectin, pullulan, starch, and xylan (González and Weiner 2000). Myt-1 was found to be capable of degrading the same polysaccharides (Table 1). Although González and Weiner (2000) reported that strain 2–40 could not degrade inulin, Myt-1 strain was capable of degrading inulin in our experiments. Moreover, strain 2–40 could not degrade chitin and pectin, but strain Myt-1 could degrade them.

**Table 1:** Extracellular polysaccharide-degrading activities of strains Myt-1 and 2–40

	Myt-1	2–40†	2–40‡
Alginic acid	+	+	+
CMC	+	+	+
Chitin	+	−	+
Fucoidan	+	±	+
Laminarin	±	±	+
Carrageenan	+	±	+
Pectin	+	−	+
Xylan	+	+	+
Polygalacturon	−	−	−
Glycogen	±	+	+
Pullulan	±	+	+
Inulin	±	−	−
Starch	+	+	+
Agar	+	+	+
Agarose	+	+	+

+, Degraded; ±, slightly degraded; –, not degraded.

† Results of this experiment.

‡ González J. M., and R. M. Weiner. 2000. Phylogenetic characterization of marine bacterium strain 2-40, a decomposer of complex polysaccharides. Int. J. Syst. Evol. Microbiol. 50:831-834.

### Detection of alginate lyase, cellulase, and agarase

**Figure 5:**
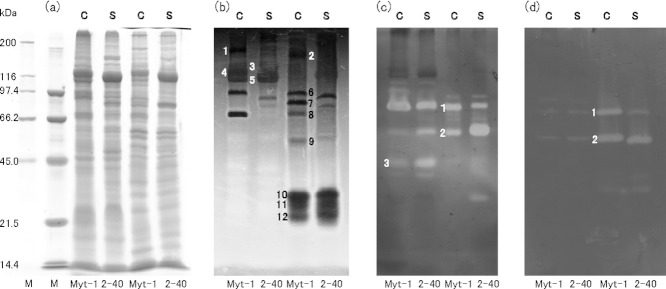
Results of sodium dodecyl sulfate-polyacrylamide gel electrophoresis and zymogram analysis of alginate lyase, cellulase, and agarase. Lane C: Whole bacterial cells obtained as a pellet by centrifugation; Lane S: culture supernatant proteins concentrated ca. 200-fold with an ultra filtration membrane. (a) Coomassie blue staining; (b) active staining for alginate lyase; (c) active staining for cellulase; (d) active staining for agarase. Major bands in Myt-1 lanes were numbered. M, molecular weight markers.

**Figure 6:**
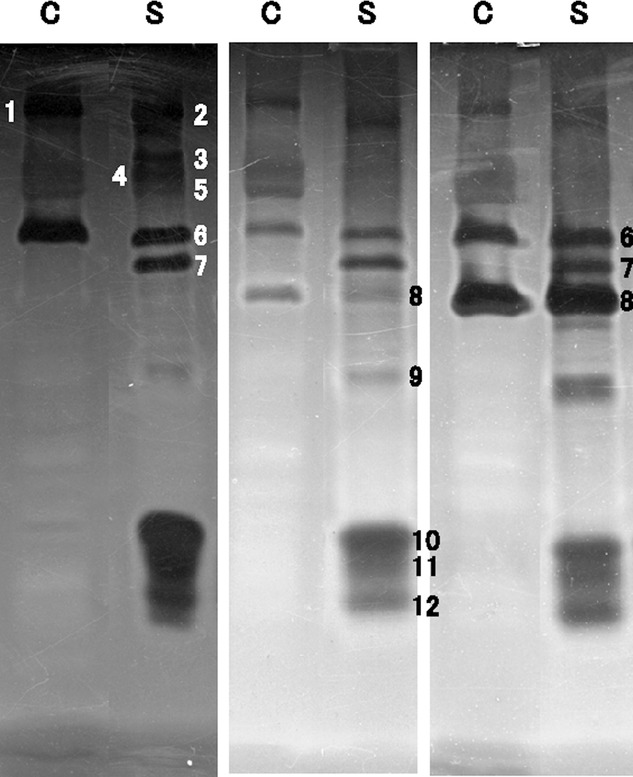
Three typical results of zymogram analysis for alginate lyases in Myt-1. These samples were obtained from the same culture conditions as those in Figure 5. Lane C: Whole bacterial cells; Lane S: culture supernatant proteins concentrated ca. 200-fold with an ultra filtration membrane.

To detect and compare the activities of the enzymes capable of degrading the main polysaccharides present in seaweeds, that is, alginate lyase, cellulase, and agarase, the enzymes in bacterial cells and in concentrated culture supernatants of strains Myt-1 and 2–40 were analyzed by SDS-PAGE and zymogram analysis (Fig. 5). Although whole protein banding patterns of strain Myt-1 were relatively similar to those of strain 2–40, numerous bands exhibited different mobilities (Fig. 5a). Although alginate lyases were detected in the cells and culture supernatants of both strains, the number of active bands and their molecular masses differed between strains (Fig. 5b). In addition, although the number and thickness (quantity or activity) of these active bands varied between experiments (Fig. 6), three prominent bands (No. 10, 11, 12 in Figs. 5b, 6) were consistently detected in the culture supernatants of both strains. Although the number of cellulases detected in both strains was similar, some of the active bands differed quantitatively from each other (Fig. 5c), and the molecular masses of the cellulases in Myt-1 were slightly lower, about 1 kDa, than those of 2–40 (No. 1, 2 in Fig. 5C). Agarases were detected, albeit faintly, in cells, and two bands exhibiting agarase activity were clearly observed in culture supernatants (Fig. 5d). The molecular masses of Myt-1 agarases were slightly higher, approximately 2 kDa, compared to those of strain 2–40 (No.1, 2 in Fig. 5d).

### Sequencing of two alginate lyase genes algMytA and algMytB

**Figure 7:**
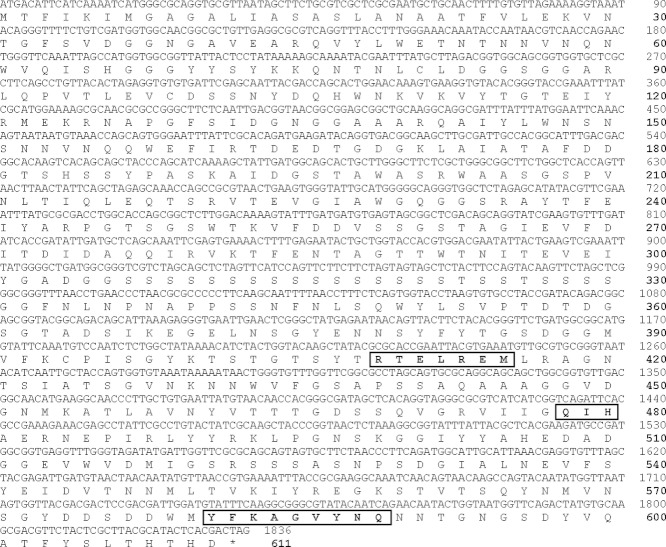
Nucleotide and deduced amino acid sequences of the gene, *algMytB*, encoding an alginate lyase. Numbering of nucleotides and amino acids (bold) is shown on the right. Start and stop codons are shown in bold. Amino acid sequences that are highly conserved among G-block–degrading enzymes in PL family 7 are boxed, and identical amino acid residues are shown in bold letters.

Two single ORFs, with lengths of 4662 bp and 1833 bp, were found in the genomic DNA of strain Myt-1 using primers designed from alginate lyase genes *alg7A* and *alg7B* of strain 2–40. These ORFs were designated as *algMytA* and *algMytB* genes, respectively. The deduced product of the *algMytA* gene is a protein of 1554 amino acids, which has an estimated molecular mass of 163.2 kDa and a pI of 4.41 (data not shown). Similarly, the deduced product of the *algMytB* gene is a protein measuring 611 amino acids with an estimated molecular mass of 65.4 kDa and a pI of 4.82 (Fig. 7). The DNA sequence and encoded protein homology of *algMytA* and *alg7A* from strain 2–40 were 97.3% and 98.3%, respectively, and those of *algMytB* and *alg7B* from strain 2–40 were 94.4% and 99.0%, respectively. The DNA sequences of the alginate lyase genes *algMytA* and *algMytB* were deposited in GenBank under accession numbers AB647186 and AB639773, respectively.

## Discussion

Numerous studies have been reported to date describing the ability of bacteria to degrade seaweed polysaccharides, such as alginate (Wong et al. 2000), laminarin (Alderkamp 2007), fucoidan (Holtkamp et al. 2009), cellulose (Taylor et al. 2006), agar (Fu and Kim 2010), carrageenan (Michel et al. 2006), and starch (Horikoshi 1999). Among these bacteria, *S. degradans* 2–40 (formerly *Microbulbifer degradans* strain 2–40), which was isolated from decaying salt marsh cordgrass in Chesapeake Bay by Andrykovitch and Marx in 1988, has been reported to degrade an extraordinary wide variety of polysaccharides (Ekborg et al. 2005; Taylor et al. 2006). Since then, detection and analysis methods for polysaccharide lyase genes and their products in strain 2–40 have progressed rapidly, and more than 20 reports have been published to date. Interestingly, however, although the entire genome of this strain has been completely sequenced (Weiner et al. 2008), no reports of any new S*accharophagus* spp. or *S. degradans* strains have been published to date. This has meant that until this report, the genus *Saccharophagus* has only contained one genus, species, and strain, that is, *S. degradans* 2–40.

We isolated the seaweed-degrading Myt-1 strain from marine sediments collected in Toyama Bay, Toyama Prefecture, Japan. To our knowledge, this is the first report to directly show that one bacterial strain is capable of decomposing several types of seaweeds (i.e., red, green, and brown algae). 16S rRNA gene sequence of strain Myt-1 was 100% identical to that of *S. degradans* 2–40. In addition, cell morphology (particularly gross surface characteristics), cell motility, and colony color changes were all very similar to those of 2–40 (Ekborg et al. 2005). Although 2–40 was unable to degrade several polysaccharides that were degraded by Myt-1 in our experiments, the ability to degrade polysaccharides was relatively similar between Myt-1 and 2–40. On the other hand, the protein banding patterns on the SDS-PAGE gels and several active alginate lyase bands on the zymograms were conspicuously different. Moreover, compared to *alg7A* and *alg7B* in 2–40, the alginate lyase genes *algMytA* and *algMytB* had lower DNA sequence and deduced amino acid sequence similarities. Combined with DNA–DNA hybridization analysis (Rossello-Mora and Amann 2001), these results strongly suggest that Myt-1 is a new *Saccharophagu*s species (Table 2), tentatively designated as *Saccharophagus* sp. Myt-1. In addition, the isolation of strain 2–40 in the United States and Myt-1 in Japan imply that the genus *Saccharophagus* has a wide geographical distribution in marine environments.

**Table 2:** Similarities and differences between Myt-1 and 2–40

	Similarities
16S rDNA	100%
DNA–DNA hybridization	65–68%
*alg MytA*	*alg 7A* in 2–40
DNA sequence	97.3%
Amino acid sequence	98.3%
*alg MytB*	*alg 7B* in 2–40
DNA sequence	94.4%
Amino acid sequence	99.0%

	Differences
Alginate lyases on Zymogram	Band No. 6, 7, 8: only detected in Myt-1*
Cellulases	Band No. 1, 2: slightly lower molecular weight (∼1 kDa) in Myt-1*
On zymogram	
Agarases	Band No. 1, 2: slightly higher molecular weight (∼2 kDa) in Myt-1*
On zymogram	

* From Figure 5.

Zymogram analysis revealed that *Saccharophagus* sp. Myt-1 possessed several catalytically active polypeptides that were capable of degrading alginate, cellulose, and agar, suggesting the existence of complex enzymatic systems for efficiently degrading these polysaccharides in this strain. Although several studies have reported the existence of similar polysaccharide lyases in *S. degradans* 2–40 (González and Weiner 2000; Ekborg et al. 2006; Taylor et al. 2006; Weiner et al. 2008; Hutcheson et al. 2011), most of the lyases in 2–40 were detected by DNA sequencing and were considered to be putative lyases. Consequently, this is likely the first report to directly demonstrate the existence of these enzymes and estimate their molecular masses in *Saccharophagus* species by SDS-PAGE and zymogram analysis. This study is considered to be important for surveying the expression of genes and to analyze those control mechanisms. The band thickness (quantity or activity) associated with the active bands of the lyases also differed between Myt-1 and 2–40, implying that the expression levels or activities of the various lyases were also different. Interestingly, when *Saccharophagus* sp. Myt-1 was cultured with Wakame thallus fragments, the number and thickness of the active bands of the alginate lyases were sometimes observed to vary (Fig. 6). On the other hand, the number and thickness of the active cellulase and agarase bands remained constant. This variation in band number and thickness is considered to have arisen because the expression of alginate lyases is unstable, and also because several alginate lyase genes of are transposable. Hutcheson *et al.* (2011) reported that there were 13 putative alginate lyases, 21 cellulases, and six agarases in strain 2–40. However, the number of bands detected in the zymogram analysis of this study was less than the number of putative polysaccharide lyases, possibly reflecting the existence of a number of complex regulatory mechanisms involved in the expression of these lyases, and also that Myt-1 may have both additional and/or novel polysaccharide lyases. To clarify this assumption in further investigations, analyses such as two-dimensional electrophoresis or the cloning of polysaccharide lyase genes, are required.

The deduced amino acid sequences of *algMytA* and *algMytB* revealed the three conserved regions that are typical of PL family 7; R in the N-terminal region of RTELREM, Q and H in loop 2, and Y in the C-terminal region of YFKAGVYNQ (Uchimura et al. 2010) (http://www.cazy.org/%Polysaccharide-Lyases.html). Because these regions are considered to be involved in the catalytic domain (Osawa et al. 2005), it is possible that the repetitive serine-rich sequence in the algMytB protein acts as a flexible linker between the catalytic and binding domains (Howard et al. 2004).

When *Saccharophagus* sp. Myt-1 cells were cultured in the presence of Wakame thallus fragments, CFU increased together with an increase in reducing sugar content and polysaccharide lyase activity. These findings suggest that strain Myt-1 decomposed Wakame thalli fragments rapidly and that the reducing sugars were used to support cell growth and related metabolic activities. On the other hand, the gradual increase in cell death observed after reaching the stationary phase was associated with a decrease in both CFU and alginate lyase activity over time. However, because sufficient quantities of reducing sugars and alginate lyase remained to sustain cell growth, it is possible that cell death may have been attributed to changes in other nutrient factors.

It has been suggested that the enzymes released by strain Myt-1 could be applied to the reduction of seaweed waste and to the production of small, functional oligosaccharides (Wong et al. 2000). It is possible that the numerous small vesicles observed on the surface of the bacterial cells and in the culture medium are related to enzyme secretion. Ekborg et al. (2005) also reported these vesicles and surface protuberances in strain 2–40. The vesicles initially formed on the cell surface from which it appears they were shed into the medium. If subsequent investigations reveal that these vesicles contain polysaccharides lyases, then it is likely that this secretion mechanism could be exploited to produce and harvest polysaccharide lyases from Myt-1. Zhang et al. (2011) recently reported the discovery of two cytoplasmic cellobiose/cellodextrin phosphorylases in *S. degradans* 2–40. Further investigations are therefore required to evaluate these enzyme activities, as well as the expression and secretion mechanisms associated with these enzymes in the future.
